# Study on the metabolic effects of hexavalent chromium [Cr (VI)] on rat astrocytes using un-targeted metabolomics

**DOI:** 10.3389/fmolb.2024.1372783

**Published:** 2024-07-05

**Authors:** Hongge Tang, Kunyang Li, Lin Lin, Wenying Wang, Wenjie Jian

**Affiliations:** ^1^ Department of Public Health and Medical Technology, Xiamen Medical College, Xiamen, Fujian, China; ^2^ Xiamen Haicang Hospital, Xiamen, Fujian, China; ^3^ Scientific Research Management Department, Brain Hospital of Hunan Province, The Second People’s Hospital of Hunan Province, Changsha, Hunan, China

**Keywords:** hexavalent chromium, astrocytes, metabolomics, oxidative damage, sphingosine, methionine

## Abstract

**Introduction:** Hexavalent chromium [Cr (VI)] has been identified as a human carcinogen and environmental pollutant capable of affecting multiple systems in the human body. However, the specific mechanisms by which Cr (VI) affects the human nervous system remain unclear.

**Objective:** Following confirmation of Cr (VI)’s toxic effects on rat astrocytes, this study explores the metabolites and associated metabolic pathways of rat astrocytes under different doses of Cr (VI) exposure.

**Methods:** Cell viability was assessed using CCK8 assays, intracellular reactive oxygen species (ROS) levels were measured using DCFH-DA fluorescent probes, intracellular 8-hydroxydeoxyguanosine (8-OHdG) content was determined by Elisa, mitochondrial membrane potential was observed using JC-1 probes, and key metabolites were identified through untargeted metabolomics analysis.

**Results:** With increasing Cr (VI) doses, significant decreases in cell viability were observed in the 4, 8, and 16 mg/L dose groups (*p* < 0.05). Elevated levels of ROS and 8-OHdG, increased caspase-3 activity, and significant reductions in mitochondrial membrane potential were observed in the 2 and 4 mg/L dose groups (*p* < 0.05). Untargeted metabolomics analysis revealed Cr (VI)’s impact on key metabolites such as sphingosine and methionine. Enrichment analysis of KEGG pathways highlighted the critical roles of sphingolipid metabolism and the methionine-cysteine cycle in the effects of Cr (VI) on rat astrocytes.

**Conclusion:** Our study underscores the potential neuro-health risks associated with environmental and occupational exposure to Cr (VI) and provides new perspectives and directions for investigating neurotoxic mechanisms.

## 1 Introduction

Chromium (Cr) is widely distributed in nature, with chromium (VI) having a more pronounced impact on humans, particularly during occupational exposure. Inhalation of Cr (VI) elevates the incidence and risk of lung cancer (Behrens et al., 2023), while ingestion via drinking water raises the risk of liver cancer (Li et al., 2023). Consequently, Cr (VI) poses risks to all bodily systems (Wang and Yang, 2019; Kouokam and Meaza, 2022), and its detrimental effects on the nervous system have garnered attention in recent years. Numerous studies have reported considerable levels of hexavalent chromium present in different brain regions among individuals exposed to it (Höck et al., 1975; Kanabrocki et al., 1975; Calderón-Garcidueñas et al., 2013). However, our understanding of the neurological health implications of Cr (VI) exposure is still nascent, and further investigation into the mechanisms underlying its toxic effects on the nervous system is warranted.

There are at least eight major cell types in the brain, including neurons, astrocytes, microglias, oligodendrocytes, pericytes, ependymal cells, smooth muscle cells and endothelial cells. In many studies of the toxic effects of Cr (VI) on the nervous system (Mustafa et al., 2016; Sanchez-Diaz et al., 2018; Singh and Chowdhuri, 2018), the role played by cell types in the toxicological mechanisms has not been considered. In contrast, astrocytes are the most common cells in the nervous system, providing energy to neurons within the brain, participating in the formation of the blood-brain barrier (BBB), and playing crucial roles in various life processes (Charles et al., 2015). Studies have observed that under exposure to Cr (VI), the number of surviving neurons in the gray matter of the rat cerebral cortex decreases significantly in dose and time, while the number of reactive astrocytes increases (Hegazy et al., 2021). Another study suggests that exposure to hexavalent chromium in astrocytes leads to cell apoptosis through the mitochondrial pathway (Wang et al., 2009). Therefore, it is necessary for us to further investigate the neurotoxicity induced by Cr (VI), particularly the toxic responses in astrocytes, to evaluate its sensitivity and toxicity to neurons throughout the entire process of neural development.

In populations exposed to Cr (VI), epidemiological studies have revealed a notable correlation between oxidative damage and adverse health outcomes (Xu et al., 2018; Hu et al., 2021). After entering the cell, Cr (VI) can be reduced to Cr (III) by certain proteins or intracellular antioxidants, generating reactive oxygen species (ROS) during the oxidation reaction, leading to oxidative stress (Jin et al., 2020), Studies have observed increased oxidative stress and neurotoxicity in fruit flies due to exposure to Cr(VI) (Vincent, 2019). The effects of ROS in the brain are widespread in the process leading to neurotoxicity, and are believed to contribute to mitochondrial dysfunction, autophagy dysfunction, and neurodegeneration (Dasuri et al., 2013; Singh and Chowdhuri, 2018; Michalska and León, 2020). Additionally, Cr (VI) can also act on mitochondria, leading to mitochondrial toxicity, which ultimately results in oxidative damage (Xiao et al., 2014). However, the exact mechanisms by which Cr (VI) exposure induces neurotoxicity remain unclear.

Metabolomics technology enables a more comprehensive assessment of the impact of environmental toxicants on the human body, providing information about relevant changes in gene and protein expression during ongoing processes (Maffezzini et al., 2020; Murat and García-Cáceres, 2021). Given the crucial role of the metabolome in various aspects of human health, elucidating the impact of Cr (VI) exposure on the metabolome and its molecular functions is necessary. Multiple research endeavors have established the involvement of Cr (VI) in carbohydrate, lipid, and nucleic acid metabolism, along with its role in transcriptional regulation (Sadeghi et al., 2015; Tian et al., 2018; Rager et al., 2019; Chen et al., 2022). Upon entry into the human body, Cr (VI) is capable of triggering oxidative damage reactions, potentially leading to metabolic dysregulation within cells.

In this study, we aimed to investigate the impact of Cr (VI) on the metabolism of astrocytes. Firstly, we assessed the toxicity of Cr (VI) on astrocytes. Subsequently, we explored the relevant mechanisms underlying Cr (VI)-induced astrocyte toxicity using untargeted metabolomics methods by UHPLC-Q-TOF-MS/MS. Our aim was to investigate, for the first time, the dynamic metabolic changes of astrocytes induced by different doses of Cr (VI) in an *in vitro* model, thus laying the groundwork for future treatment and prevention strategies.

## 2 Materials and methods

### 2.1 Chemical and regents

Hexavalent chromium Standard solution (Cat. No: 19073) was purchased from China Institute of Metrology, Beijing, China. Dulbecco’s Modified Eagle’s Medium (DMEM, Cat. No: c3110-0500) and fetal bovine serum (FBS, Cat. No: c04001-500) were purchased from Biological Industries, Israel. The JC-1 mitochondrial membrane potential assay kit and 2′,7′-Dichlorodihydrofluorescein diacetate (DCFH-DA) probe were sourced from Beyotime in Beijing, China. The grades of methanol, formic acid, and acetonitrile are all analytical, which were purchased from Sigma, United States.

### 2.2 Cell culture and treatment

**FIGURE 1:**
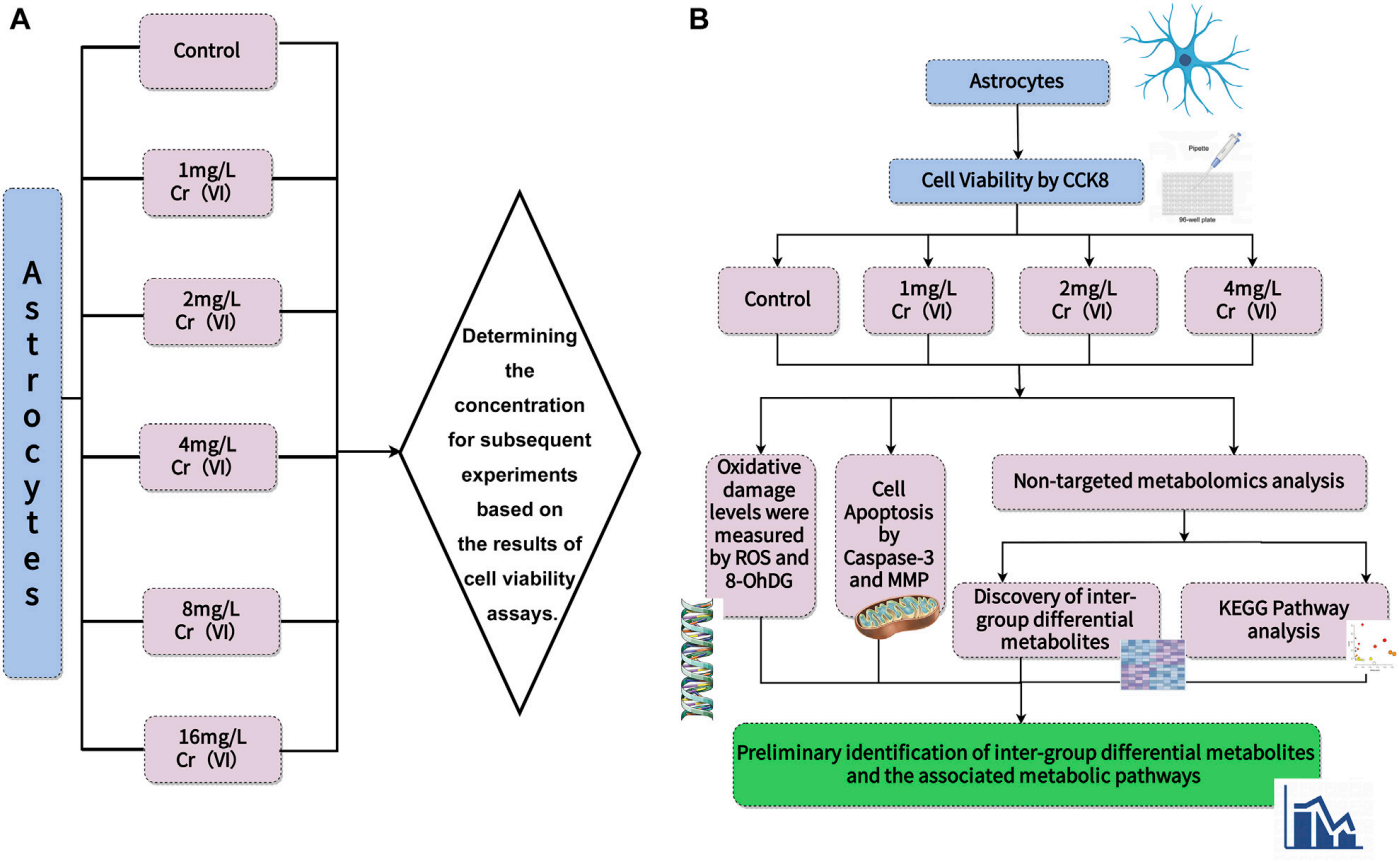
The scheme of experiments and measurements. **(A)** Cell viability was determined to establish concentrations. **(B)** Different concentrations were used to measure ROS levels and 8-OHdG content to observe oxidative damage. Next, Caspase-3 activity was measured, and changes in mitochondrial membrane potential were depicted using images to describe cell apoptosis. Finally, UHPLC-Q-TOF-MS/MS was employed for untargeted measurement of metabolite changes and to infer metabolic pathways.

CTX-TNA2 rat brain astrocyte cell line was obtained from Shanghai Gaining Type Culture Collection (Shanghai, China). The cells were cultured (37°C, 5% CO_2_) in DMEM supplemented with 10% heat-inactivated FBS and 1% penicillin-streptomycin. After reaching the confluence, cells were detached by using 0.05% (w/v) trypsin/EDTA for 5 min at 37°C. Hexavalent chromium Standard solution (100 mg/L) was prepared as a stock solution with double distilled water and diluted into a final concentration, before being used with DMEM containing 10% FBS. When the cell density reached 5 × 10^6^ cells per 100 mm dish, the cells were treated with 0, 1, 2, 4, 8, 16 mg/L for 24 h to determine the effect of different Cr(VI) concentrations. In addition, the control group cells were cultured in 10% FBS without any treatment for an additional 24 h, and then collected for the following determination. All the experiments and measurements are presented in Figure 1.

### 2.3 Cell proliferation assay

As per the manufacturer’s instructions, the proliferation of astrocytes was evaluated using the Cell Counting Kit-8 (CCK-8, Solarbio, China, Cat. No: MAB0218). Briefly, 2 × 10^3^ cells/well were seeded into 96-well plates, and 10 µL of CCK-8 solution was added to each well along with 90 µL of DMEM medium. The final concentration of CCK8 is 10%. The plates were then incubated at 37°C with 5% CO_2_ for 24 h. Absorbance at 450 nm was measured using a micro-plate reader (Infinite 200, Tecan, Switzerland).

### 2.4 Assessment of intracellular ROS levels

DCFH-DA (DCFA) is a fluorescent probe commonly used to measure the levels of ROS within cells. DCFA can enter cells and undergo deacetylation to form 2′, 7′-Dichlorodihydrofluorescein (DCFH), which reacts with intracellular ROS to generate the highly fluorescent product 2′,7′-Dichlorofluorescein (DCF). By measuring the fluorescence intensity of DCF, the intracellular levels of ROS can be indirectly assessed, enabling the study of oxidative stress and cellular oxidative status. In this study, cells were incubated in with 10 μM DCFA (Cat. No: S0033S, Beyotime, Beijing, China) dissolved in 0.01 M PBS at 37°C for 30 min, and washed twice with 0.01 M PBS at 37°C. Then, the relative fluorescence intensity of DCFA in the cells was measured at an excitation wavelength of 480 nm and an emission wavelength of 535 nm by a fluorescence micro-plate reader (Infinite 200, Tecan, Switzerland).

### 2.5 Assessment of 8-OHdG

The concentration of 8-OHdG in the lysates of rat astrocytes was measured using a rat-specific 8-OHdG ELISA kit (Shanghai Meilian Biological Technology Co., Ltd., Shanghai, China, Cat. No: ML077380) according to the manufacturer’s protocol. The ELISA kits have an intra-assay coefficient of variation of less than 10% and an inter-assay coefficient of variation of less than 15%.

### 2.6 Assessment of Caspase-3 activity

As per the manufacturer’s instructions, Caspase-3 activity was assessed using the Caspase-3 Activity Assay Kit (Solarbio, Beijing, Cat. No: BC3830). Activated Caspase-3 can specifically cleave the DEVD-X substrate, hydrolyzing the D4-X peptide bond. Based on this characteristic, a fluorescently labeled short peptide Ac-DEVD-AMC is designed. During covalent coupling, AMC cannot be excited to emit fluorescence. After hydrolysis of the short peptide, AMC is released, and only free AMC can be excited to emit fluorescence. The intensity of the released AMC fluorescence can be used to measure the activity of caspase-3, thereby reflecting the degree of Caspase-3 activation. At the conclusion of the exposure period, astrocytes were collected by scraping. Briefly, 1 × 10^6^ cells were lysed in 50 μL lysis buffer on ice for 10 min, followed by centrifugation at 12,000 *g* at 4°C for 10 min to collect the supernatant. The optical density of the samples was measured using a microplate reader (Infinite 200, Tecan, Switzerland) at 405 nm.

### 2.7 Assessment of mitochondrial membrane potential (ΔΨm, MMP)

MMP represents the electrical potential difference across the inner mitochondrial membrane. It is a measure of the energy stored in the form of electrochemical gradient across the membrane, which is critical for various cellular processes, including ATP production, ion transport, and regulation of cell survival and apoptosis.

The MMP analysis was conducted using the JC-1 mitochondrial membrane potential assay kit (Beyotime, Beijing, China, Cat. No: c2001s). In brief, astrocytes (2 × 10^5^) were seeded in 6-well plates and, following appropriate drug treatments, stained with 10 μM JC-1 for 20 min at 37°C. Red fluorescence signified the accumulation of JC-1 in the mitochondria in a manner dependent on the membrane potential. Conversely, green fluorescence indicated the presence of the monomeric form of JC-1 in the cytosol. Fluorescence images were captured under an Olympus IX71 fluorescence microscope, and images were acquired using an Olympus SC35 digital camera system.

Subsequently, this study quantified the MMP levels using the TMRE probe (Beyotime, C2001). 5 × 10^3^ cells were seeded in each well of a 96-well plate and incubated with TMRE for 45 min at 37°C. Fluorescence intensity was measured using a multifunctional microplate reader (excitation = 545 nm and emission = 590 nm).

### 2.8 Metabolomics analysis

#### 2.8.1 Sample preparation

At the conclusion of the exposure period, astrocytes were collected by scraping. Each sample was then added to 400 µL of methanol and thoroughly mixed by vortexing for 30 s. Subsequently, low-temperature ultrasonic extraction was performed for 30 min at 5°C and 40 kHz. The samples were then cooled to −20°C for 30 min, followed by centrifugation at 4°C for 15 min at 1000 rpm. The supernatant was extracted for testing, while the pellet was dried using nitrogen gas. To the centrifuge tube containing the dried cell sample, 400 µL of precooled methanol was added. The cells were re-dissolved by thorough vortexing, and the centrifuge tube was placed in liquid nitrogen for 2 min to achieve complete freezing. After removing the tube from liquid nitrogen, it was agitated in an ice water bath for 2 min to fully dissolve the contents. This freeze-thaw process was repeated 4 times, followed by high-speed centrifugation (12,000 rpm, 10 min) at 4°C to complete the procedure, and all supernatants were mixed for testing. Each group for cellular metabolomics analysis consisted of a minimum of six replicates.

#### 2.8.2 Ultra-HPLC coupled with quadrupole-TOF-MS/MS profiling analysis

An Agilent 1290 Infinity II LC system coupled with an Agilent 6545 Accurate Mass Quadrupole Time-of-Flight mass spectrometer (Agilent Technologies Inc., CA, United States) was employed for Ultra-HPLC analysis. Chromatographic separations were conducted at 40°C on a reversed-phase C18 column (150 × 2.1 mm, 1.8 μm particle size, Agilent, CA, United States). The mobile phase consisted of 0.1% formic acid (A) and acetonitrile modified with 0.1% formic acid (B). The optimized UPLC elution condition was as follows: 5% MPB at t = 0 min, 30% MPB at t = 6 min, 80% MPB at t = 15 min, 95% MPB at t = 25 min, 5% MPB at t = 25.1 min, and 5% MPB at t = 30 min. The flow rate was set to 0.4 mL/min, and the injection volume was 10 μL. The auto sampler was maintained at 4°C. An Agilent jet stream source (AJS) ESI source was utilized in positive modes. The AJS ESI conditions included nitrogen drying gas at a flow rate of 8 L/min and a temperature of 325°C, a nebulizer gas (nitrogen) pressure of 35 psi, sheath gas flow rate of 12 L/min, sheath gas temperature of 400°C, frag mentor voltage of 130 V, skimmer voltage of 45 V, capillary voltage of 3500 V in a positive mode, and nozzle voltage of 1000 V.

### 2.9 Statistics

All of the values are presented as mean ± SD of at least four independent experiments and were analyzed by the SPSS software for windows, v20.0 (SPSS Inc., United States). Significant differences among group means were evaluated by analysis of variance test (one-way ANOVA). Post hoc tests were analyzed by Student-Newman-Keuls (SNK) test. The significance was defined as *p* < 0.05.

The UHPLC-Q-TOF-MS/MS raw data files were processed for peak detection and alignment using Agilent Profinder 10.0 software from Agilent Technology Co., Ltd. (Santa Clara, California, United States), to obtain a data matrix containing the m/z values, retention time, and peak intensities for each sample. Subsequently, the filtered data was matched with the METLIN (https://metlin.scripps.edu) database (metabolite spectral database) using Mass Profiler Professional software 15.1 from Agilent Technologies (Santa Clara, California, United States), with a database scoring threshold of >70 and a mass error of <10 ppm. All data have been standardized using z-score normalization.

Partial Least Squares Discriminant Analysis (PLS-DA) is a discriminant analysis method in multivariate data analysis technology, often used to handle classification and discrimination problems. By appropriately rotating the principal components, PLS-DA can effectively distinguish between group observations and identify the influencing variables that lead to inter group differences. Assessing the performance of the analytical system and identifying metabolic trends, clustering, and separation in sample metabolomes through PLS-DA. Utilizing model fit (R2X/R2Y series) and predictive ability (Q2) values (leave-one-out (LOO) cross-validation) (Mohammad et al., 2022).

The volcano plots and venn diagram of the different metabolites was performed on the MetaboAnalyst platform (http://www.metaboanalyst.ca/). An enrichment analysis was performed using MetaboAnalyst software 5.0 to identify the metabolite pathways based on Kyoto Encyclopedia of Genes and Genomes (KEGG) database.

## 3 Results and discussion

There are several new discoveries in this study. First, we demonstrated that the levels of mitochondrial membrane potential decreased, and the cellular DNA is oxidized in Cr (VI) treated rat astrocytes, and second, the sphingolipid metabolism and methionine cysteine cycle play an important role upon treatment with Cr (VI) in rat astrocytes.

Astrocytes are the largest and most important group of glial in the central nervous system (CNS), providing structural, capacity and metabolic support for neurons, and playing a regulatory role in neuronal synaptic transmission (Pathak and Sriram, 2023). These cells can also maintain the stability of pH, ions and neurotransmitters in the synaptic space (Sulimai et al., 2023). In terms of structure, these cells have an extension process called foot plate or end foot. These processes surround the surface of blood vessels in the brain, forming perivascular and piaglial restricted membranes. These cell structural characteristics make astrocytes as the first cells in the brain substance to encounter foreign molecules passing through the BBB, which shows the importance of astrocytes in maintaining the integrity of the BBB.

### 3.1 Oxidative damage and apoptosis in rat astrocytes induced by Cr (VI) treatment

**FIGURE 2:**
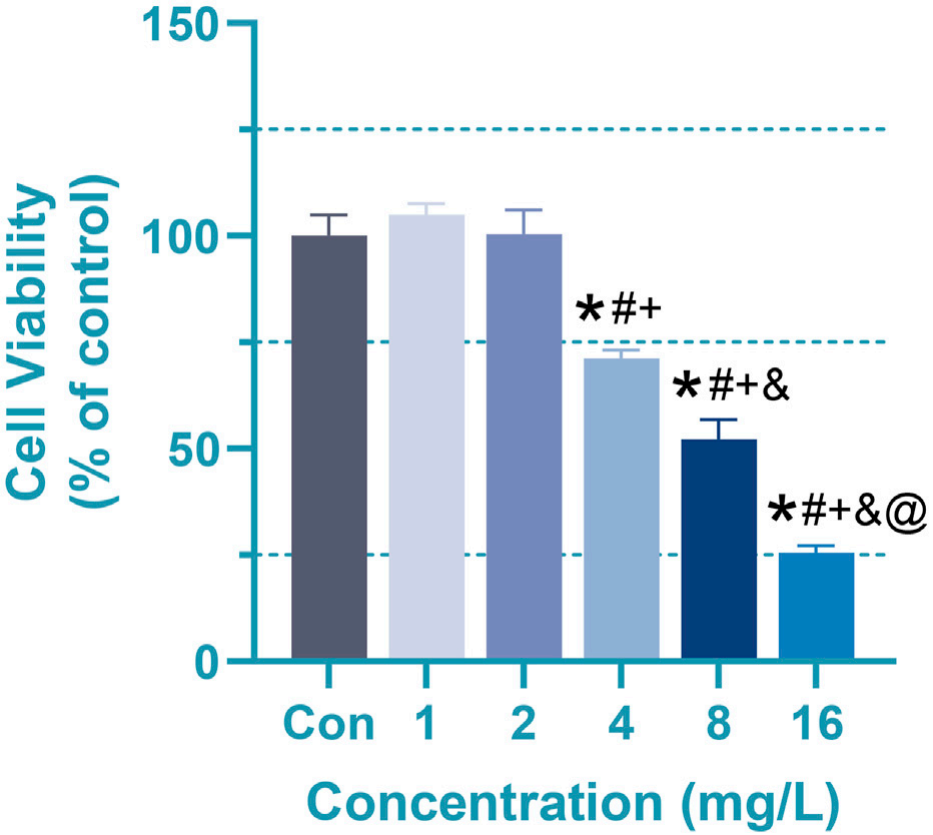
Effect of Cr (VI) on the viability of rat astrocyte. Cell viability of astrocytes measured by CCK8 assays. Cells were treated by Cr (VI) with 1 (19.2 μM), 2 (38.5 μM), 4(76.92 μM), 8 (153.86 μM) and 16 (307.72 μM) mg/mL respectively, for 24 h. Con, untreated cells. All of the values are presented as mean ± SD; n = 4; *, *p* < 0.05 compared with the control value, #, *p* < 0.05 compared with the 1 mg/L value, +, *p* < 0.05 compared with the 2 mg/L value, and, *p* < 0.05 compared with the 4 mg/L value, @, *p* < 0.05 compared with the 8 mg/L value.

CCK8 was utilized to determine the appropriate Cr (VI) concentration for further investigations, with the outcomes being presented in Figure 2. As indicated, the cell viability in 1 mg/L (19.2 μM) and 2 mg/L (38.5 μM) groups have no significant difference, whereas 4–16 mg/L (76.92–307.72 μM) Cr(VI) could considerably limit astrocyte growth in a dose-dependent manner. The mean ± SD values for the six groups are as follows: 100 ± 4.97, 104.96 ± 2.63, 100.39 ± 5.70, 71.15 ± 1.98, 52.18 ± 4.63, 25.56 ± 1.60. In order to investigate whether there are changes in metabolites or other details before significant changes occur in cell viability, for the following experiments, the astrocytes were treated with 1 (19.2 μM), 2 (38.5 μM), and 4 mg/L (76.92 μM) Cr (VI) for 24 h, respectively.

The selection of Cr (VI) concentrations was informed by existing literature on the toxic effects of hexavalent chromium on cellular systems and environmental exposure levels. The doses used in our experiments were extrapolated from both *in vivo* and *in vitro* data. For instance, levels of Cr(VI) in coastal sediments of South Korea ranged from 7.0 mg/L to 233.0 mg/L (approximately 134.6 μM–4,481.1 μM) (Lim et al., 2007). Furthermore, epidemiological studies have indicated that chromate workers exhibit micromolar concentrations (30–100 μM) of Cr (VI) in peripheral lung tissue, with even millimolar concentrations detected in the lungs within certain occupational settings (Raithel et al., 1993; Ishikawa et al., 1994). Meanwhile, in other studies, exposure to 95 mg/kg body weight of Cr (VI) has been shown to increase lipid peroxidation levels, induce significant DNA fragmentation, and enhance the generation of superoxide anions in the brain tissue of mice (Bagchi et al., 2001). Moreover, exposure to 10 μM Cr (VI) leads to a notable increase in ROS levels in cerebellar granule cells and a decrease in MMP levels in immature neurons (Dashti et al., 2016). Additionally, the viability of 16HBE cells is significantly decreased and cellular stress induced by Cr (VI) is intensified at a concentration of 12.5 μM Cr (VI) (Hu et al., 2016). Cr (VI) induces cytotoxicity and cell death in neuronal cells by triggering a mitochondrial-dependent apoptotic pathway mediated by the generation of ROS (Fu et al., 2020). Our focus was specifically on concentrations relevant to human exposure scenarios, ensuring coverage of a broad range of physiological and environmental conditions.

The selection of Cr (VI) concentrations [1, 2, and 4 mg/L (19.2, 38.5 and 76.92 μM)] is based on the principle that cell viability below 50% indicates a significant loss of cell life, representing a critical condition. High concentrations leading to such acute toxicity are rarely encountered in real-world scenarios. It is also essential to consider that even with relatively high exposure levels, the actual dose reaching the brain and nervous system is substantially reduced. Consequently, the research focuses on examining alterations in key indicators and metabolites under conditions where cell viability is largely preserved or only marginally affected. This approach mirrors typical exposure scenarios to hexavalent chromium in occupational settings, providing a relevant basis for the study.

**FIGURE 3:**
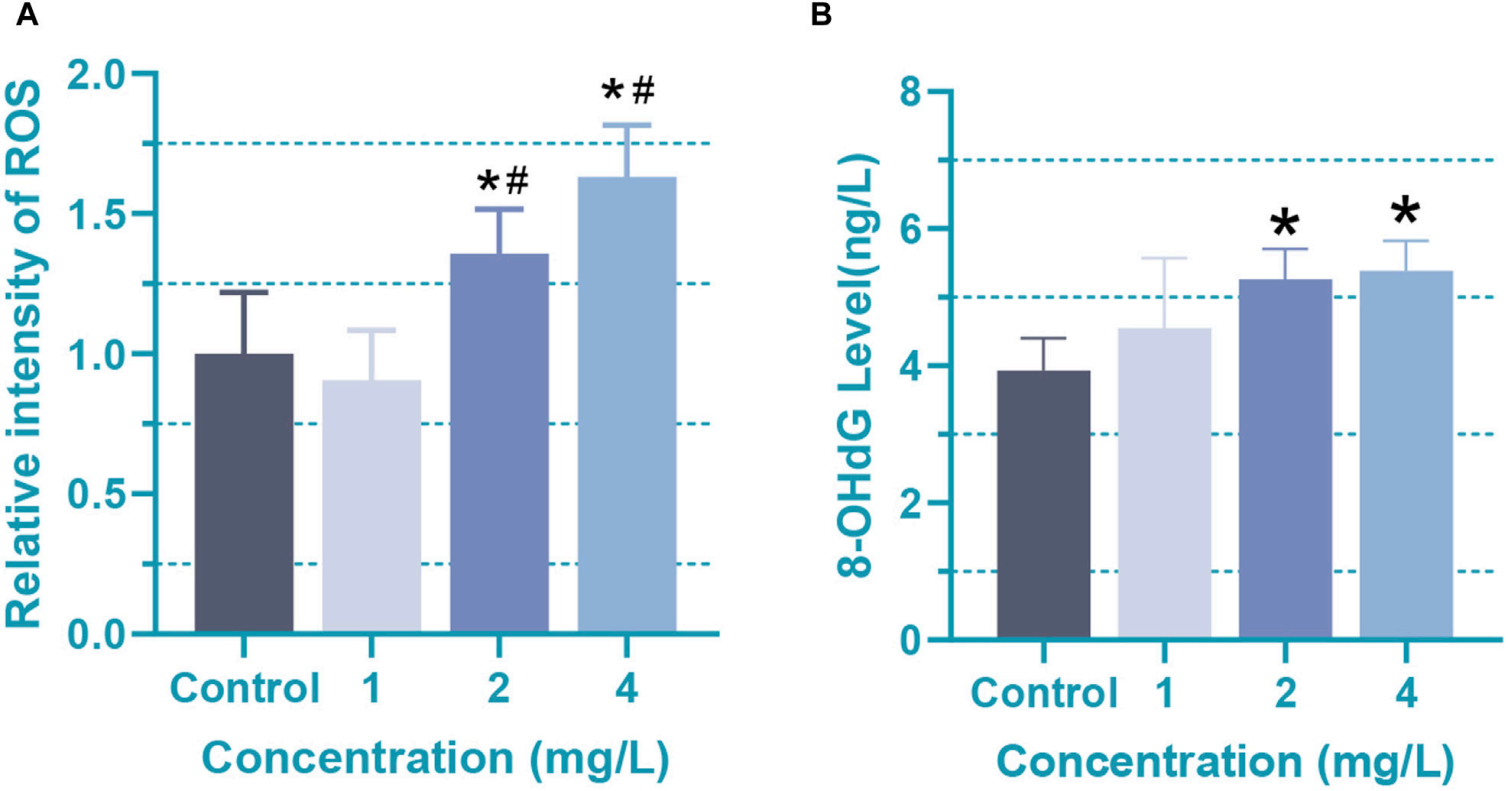
Oxidative damage in rat astrocytes induced by Cr (VI). **(A)** The ROS levels of Cr (VI) treated astrocytes. **(B)** The content of 8-OHdG in Cr (VI) treated astrocytes. Cells were treated by Cr (VI) with 1 (19.2 μM), 2 (38.5 μM), and 4 (76.92 μM) mg/mL respectively, for 24 h. Control, untreated cells. All of the values are presented as mean ± SD; n = 4; *, *p* < 0.05 compared with the control value, #, *p* < 0.05 compared with the 1 mg/L value.

Due to the imbalance between the detoxification ability of cells and the production of free radicals during the process, the cytotoxicity of Cr (VI) is closely related to the formation of reactive oxygen species (ROS) and free radicals during the reduction reaction to Cr (III) (Husain and Mahmood, 2017). High levels of ROS often lead to cell damage, chromosome breakage, and the formation of DNA adducts. Studies have shown that the toxicity of Cr (VI) can lead to the oxidation of DNA adducts, multiple copies of ribosomal DNA (rDNA), and chromosome damage, which is likely associated with the formation of ROS (Lou et al., 2021). Especially in the nervous system, a large number of studies have shown that Cr (VI) can lead to excessive ROS generation, resulting in oxidative stress events that are a condition for the generation of many neuropath logical events (Xu et al., 2021). In our study, oxidative stress and DNA damage levels in Cr (VI)-treated astrocytes were evaluated by measuring the levels of ROS and 8-hydroxyguanine (8-OHdG). A significant increase (*p* < 0.05) in ROS levels in rat astrocytes was found after 24 h of treatment with 2 (38.5 μM)and 4 mg/L (76.92 μM) Cr (VI) (Figure 3A). The mean ± SD values for the four groups are as follows: 1 ± 0.22, 0.91 ± 0.18, 1.36 ± 0.16, 1.63 ± 0.18. Correspondingly, 8-OHdG levels in cells upon treatment with 2 (38.5 μM) and 4 mg/L (76.92 μM) Cr (VI) increased markedly in a dose-dependent manner (Figure 3B). The mean ± SD values for the four groups are as follows: 3.93 ± 0.47, 4.55 ± 1.02, 5.26 ± 0.43, 5.38 ± 0.44. 8-OHdG is the product of oxidative modification in the carbon atom at the 8-position of deoxyguanosine, which is a marker of oxidized DNA (Hahm et al., 2022). Oxidation of the bases is one of the causes of damaged DNA. Together, these findings suggest that the excessive production of ROS in rat astrocytes exposed to Cr (VI) could induce DNA damage and subsequently disrupt the integrity of the mitochondrial membrane. Thus, our studies demonstrated that enhanced ROS generation might result in oxidation of mitochondrial and nuclear DNA in rat astrocytes.

**FIGURE 4:**
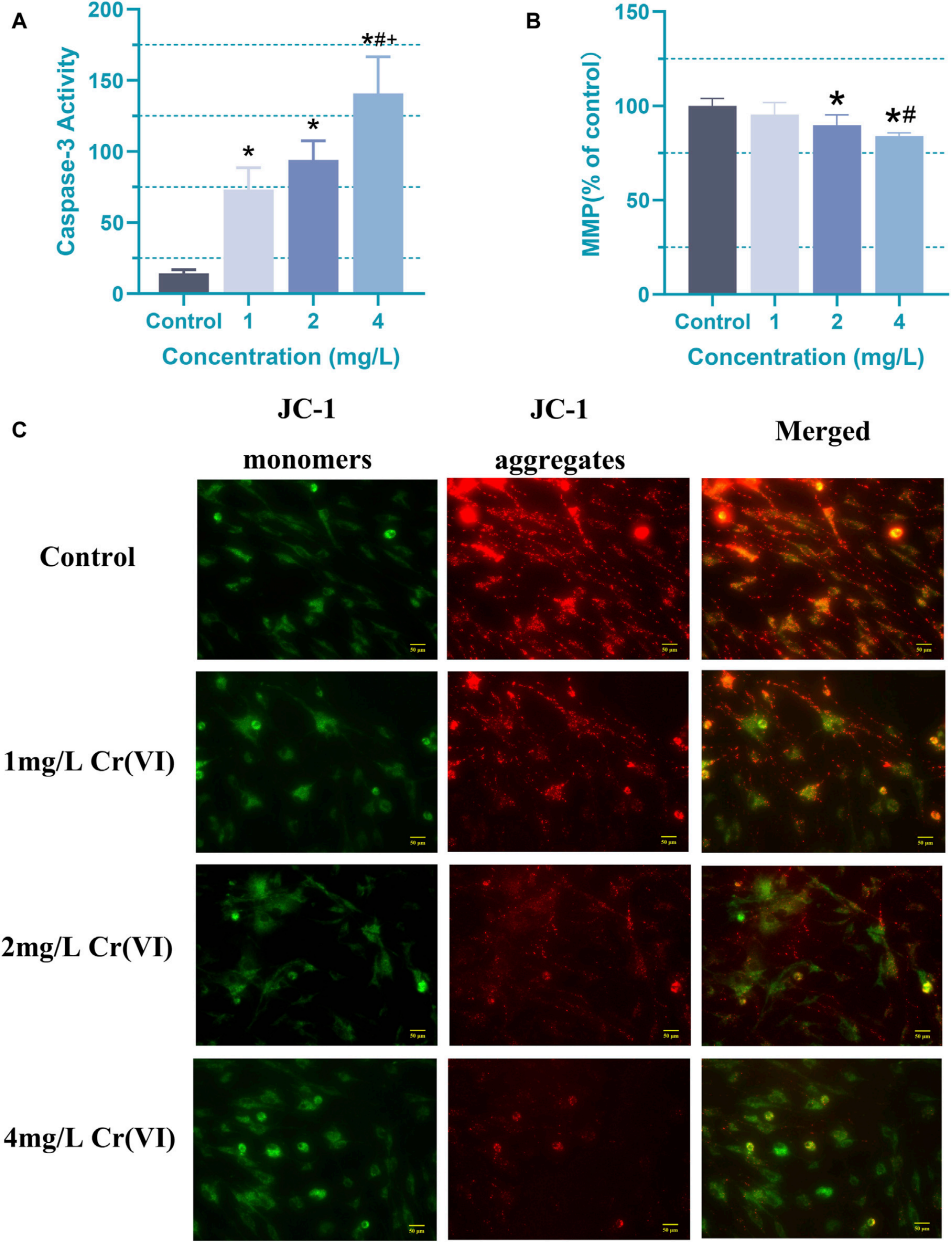
Apoptosis induced by Cr (VI) in rat astrocyte. **(A)** Changes in caspase-3 activity of astrocytes in each group. **(B)** The ΔΨm was detected by Cr (VI) in the astrocytes stained with Tetramethylrhodamine ethyl ester perchlorate (TMRE). The excitation/emission wavelengths are 545/590 nm. **(C)** Images of mitochondrial membrane potential assessed using JC-1 staining (magnification: ×400). The red color signifies the mitochondrial accumulation of JC-1, indicative of intact mitochondrial membrane potential, whereas the green color represents the monomeric form of JC-1 dispersed in the cytosol. Cells were treated by Cr (VI) with 1 (19.2 μM), 2 (38.5 μM), and 4 (76.92 μM) mg/mL respectively, for 24 h. Control, untreated cells. All of the values are presented as mean ± SD; n = 4; *, *p* < 0.05 compared with the control value, #, *p* < 0.05 compared with the 1 mg/L value, +, *p* < 0.05 compared with the 2 mg/L value.

Caspase-3, serving as the ultimate executor in caspase activation, orchestrates the final stages of nuclear events associated with apoptosis (Yeganeh et al., 2015; Martelli et al., 2022; Paskeh et al., 2022). In order to investigate whether there are changes in metabolites or other details before significant changes occur in cell viability, for the following experiments, the astrocytes were treated with 1 (19.2 μM), 2 (38.5 μM), and 4 mg/L (76.92 μM) Cr (VI) for 24 h, respectively. At the 24-h mark, we assessed their activity to examine the influence of caspase activation on apoptosis induced by 1, 2, and 4 mg/L (19.2, 38.5, and 76.92 μM) Cr (VI) in rat astrocytes. Obviously, compared to the control group, the activity of caspase-3 significantly increased in the 1 (19.2 μM), 2 (38.5 μM), and 4 (76.92 μM) mg/L groups (Figure 4A). The mean ± SD values for the four groups are as follows: 14.22 ± 2.71, 73.19 ± 15.43, 94.05 ± 13.46, 140.91 ± 25.75. At lower concentrations, there is already an observable effect on caspase-3 activity, albeit without a consequential impact on cell viability. This suggests a heightened sensitivity of caspase-3 to the toxic substance, indicating a cellular response even at minimal exposure levels. However, studies have also shown that caspase-3 plays a crucial role in tissue differentiation, regeneration, and neural development (Asadi et al., 2022). Since the cells used in the study are astrocytes derived from one-day-old rats, it is likely that many developmental pathways are still active. Meanwhile, compared with 1 (19.2 μM) and 2 (38.5 μM) mg/L Cr (VI), the 4 mg/L (76.92 μM) Cr (VI) treatment induced significantly higher caspase-3 activity (Figure 4A), while the MMP in astrocyte decreased significantly after 2 and 4 mg/L treatment (Figures 4B, C).

This study revealed that Cr (VI) treatment led to reduced astrocyte viability, elevated ROS and 8-OHdG generation, diminished mitochondrial membrane potential, and triggered apoptosis in rat astrocytes. These findings suggest the activation of an apoptotic pathway mediated by oxidative stress in astrocytes. Some studies also indicate that Cr (VI) can induce an increase in intracellular ROS and cell death in rat astrocytes through a mitochondrial-mediated pathway (Hegazy et al., 2021). Additionally, it causes neurotoxicity in rat hippocampal neurons through ROS-mediated oxidative damage (Zhang et al., 2023). We can gain preliminary insights into the apoptotic mechanisms of neuronal cells caused by Cr (VI). So we next analyzed the metabolites and metabolic pathways of astrocyte under the influence of Cr (VI), providing prevention and treatment targets for the next research.

### 3.2 PLS-DA of samples from each exposure groups and control groups

The model parameters R2X, R2Y, and Q2 are used to assess the effectiveness and predictive ability of the model. R2X represents the proportion of variance explained in the X matrix, R2Y represents the proportion of variance explained in the Y matrix, and Q2 represents the predictive ability of the model during cross-validation. Higher values of R2X and R2Y indicate that the model can effectively explain the variability in the data, while a higher Q2 value suggests better predictive performance of the model on new samples. R2Y should consistently exceed Q2, with higher values indicating better performance; ideally, R2X should exceed 0.5 (Liu et al., 2022).

**FIGURE 5:**
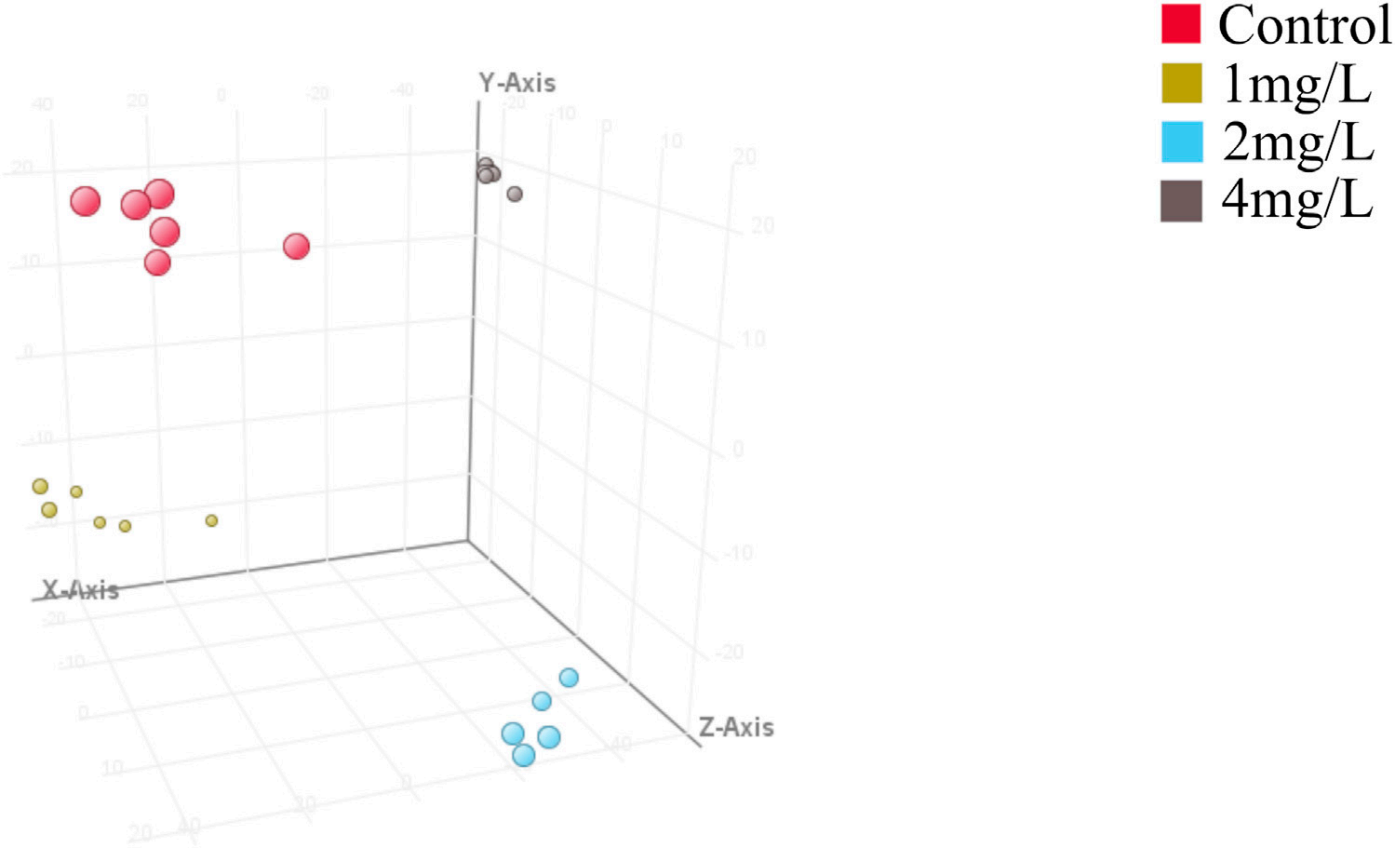
Partial least squares discriminant analysis (PLS-DA). Red dot represents the control, yellow dot represents 1 mg/L (19.2 μM) Cr (VI)-exposed group, blue dot represents 2 mg/L (38.5 μM) Cr (VI)-exposed group and grey dot represents 4 mg/L (76.92 μM) Cr (VI)-exposed group, n = 6. The model parameters in different treatment groups are shown in Table 1. Model parameters R2X, R2Y, and Q2 assess model effectiveness and predictability. R2X explains X matrix variance, R2Y explains Y matrix variance, and Q2 evaluates cross-validation predictive ability. Higher R2X and R2Y values signify better data variability explanation, while a superior Q2 indicates improved prediction on new samples. R2Y should consistently surpass Q2 for optimal performance, ideally with R2X exceeding 0.5.

Further comparative PLS-DA of each class of the differentiated CTX-TNA2 cells exposed to different Cr (VI) showed that they were clearly separated from the control (Figure 5), indicating that their metabolic profiles are distinct from one class to another. Cross-validation of these PLS-DA models was excellent with an R2Y of 0.647–0.995 and Q2 of 0.462–0.887, these values indicate that the models are robust as they are higher than the recommended values of 0.40 of R2Y and Q2 for untargeted metabolomics. These models, therefore, were used for the identification of significantly altered metabolites in the Cr (VI)-exposed samples compared to the control.

### 3.3 Identification of differentially abundant metabolites

**FIGURE 6:**
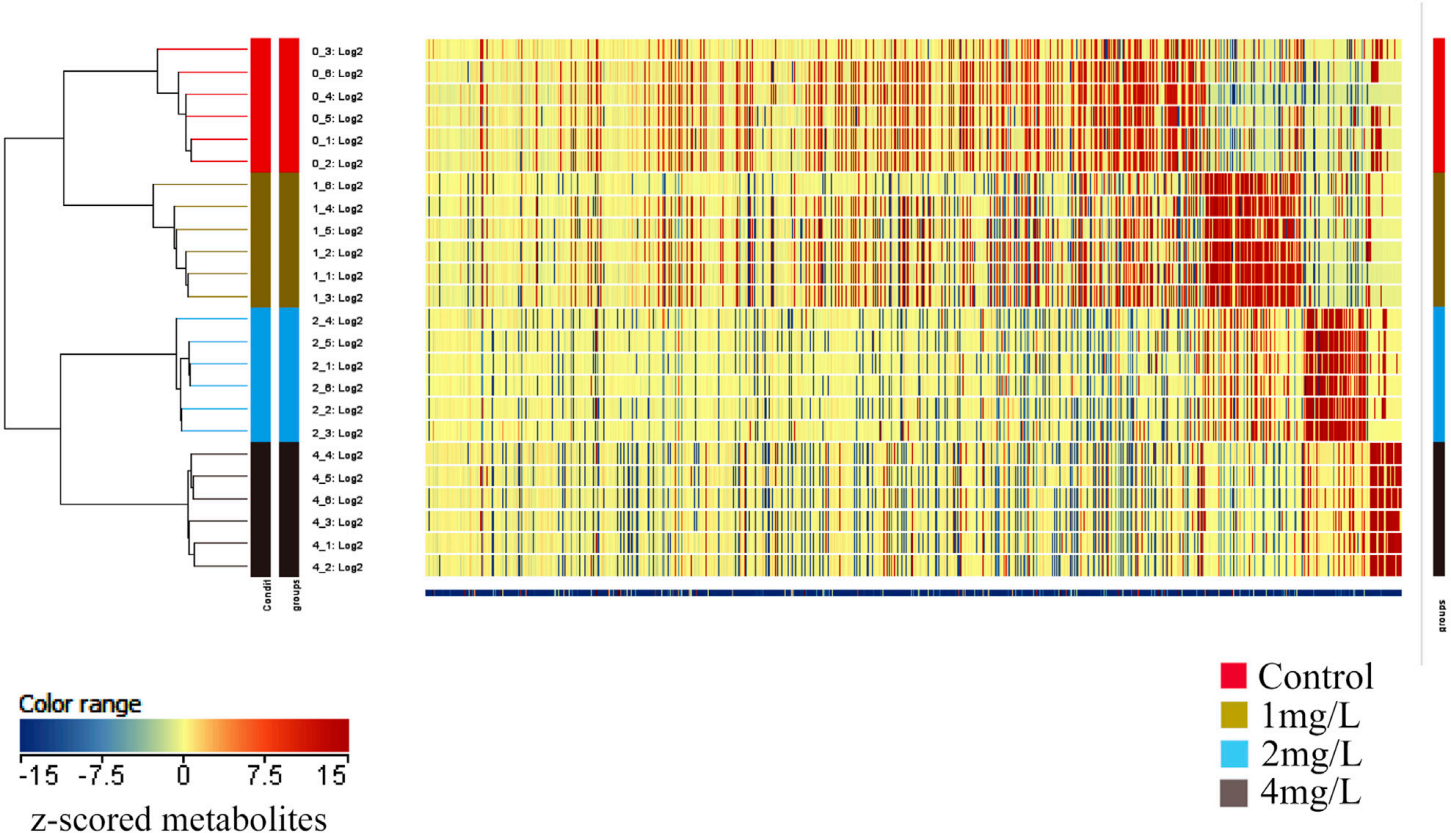
The heat map depicts the clustering of metabolites across different groups. The horizontal axis represents the sample numbers and their clustering relationships, while the vertical axis indicates the clustering relationships of metabolites. Red and blue colors denote high and low expression levels of the respective metabolites.

In order to gain deeper insights into the metabolite alterations induced by Cr (VI), both primary and secondary metabolites were identified through UHPLC-Q-TOF-MS/MS analysis. To comprehensively assess the metabolomics model, a cluster heat map was constructed. The outcomes revealed distinct classification of the 24 samples into four groups, underscoring the robustness and reproducibility of the findings (Figure 6).

**FIGURE 7:**
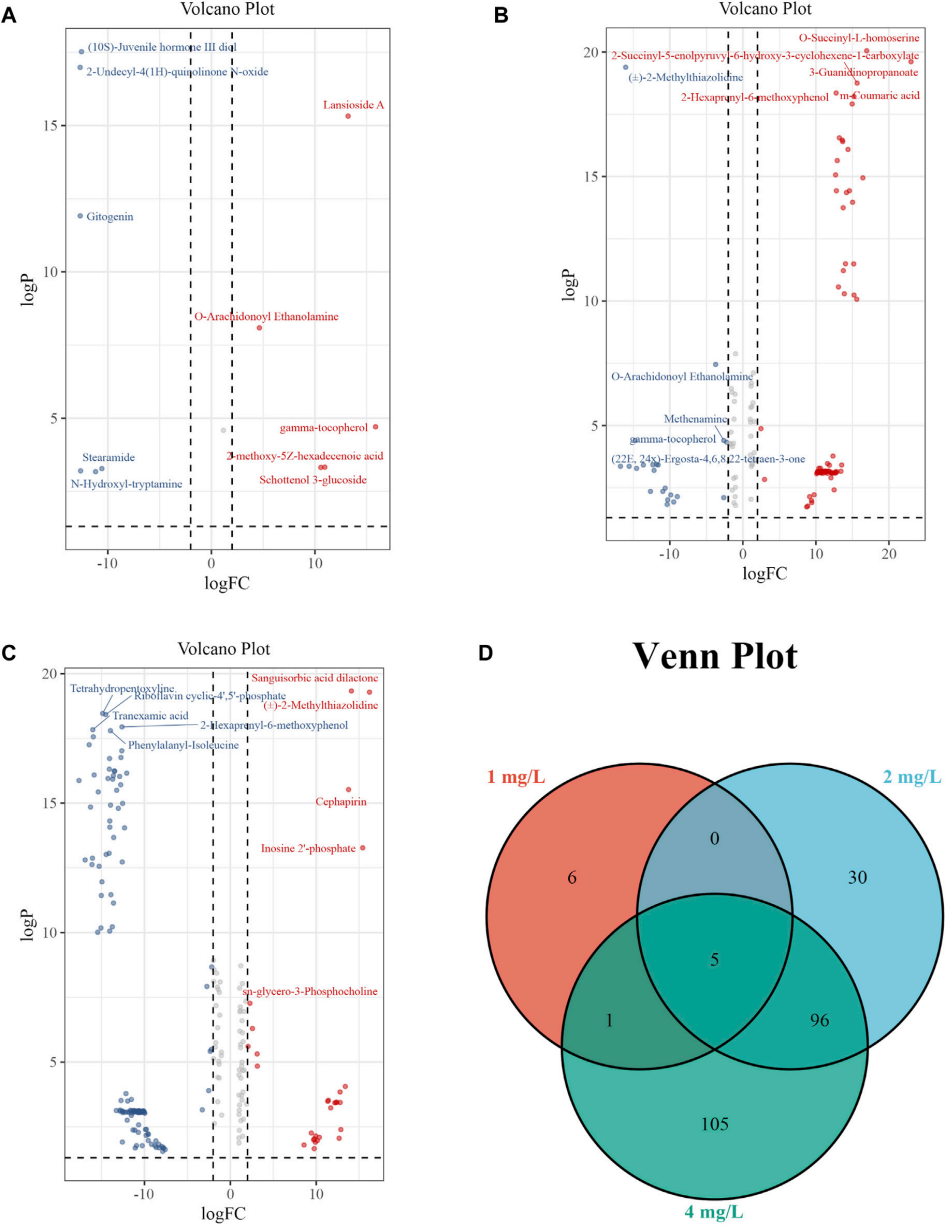
Volcano plots and Venn plot. **(A)** Volcano plot of 1 mg/L (19.2 μM) Cr (VI)-exposed group vs. Control; **(B)** Volcano plot of 2 mg/L (38.5 μM) Cr (VI)-exposed group vs. Control. **(C)** Volcano plot of 4 mg/L (76.92 μM) Cr (VI)-exposed group vs. Control. **(D)** Venn plot among 1, 2, and 4 mg/L Cr (VI)-exposed group.

The volcano plot with Log_2_FC > 2 and *p* < 0.05 displays the differential expression levels of metabolites between each pair of groups. There were 12 metabolites with differential abundance between the Control group and the 1 mg/L (19.2 μM) group, of which 6 were downregulated and 6 were upregulated in the 1 mg/L (19.2 μM) group (Figure 7A); 131 metabolites with differential abundance between the Control group and 2 mg/L (38.5 μM) group, of which 40 were downregulated and 91 were upregulated in the 2 mg/L (38.5 μM) group (Figure 7B); 208 metabolites with differential abundance between the Control group and 4 mg/L (76.92 μM) group, of which 143 were downregulated and 64 were upregulated in the 4 mg/L (76.92 μM) group (Figure 7C). The relationship among differential metabolites between groups is shown through a Venn diagram, indicating that all exposed groups share 5 metabolites, namely O-Arachidonoyl Ethanolamine, gamma-tocopherol, Cyclandelate, Stearamide, and Penicillin G (Figure 7D). The differential metabolites of each group are displayed in the Supplementary Material.

### 3.4 KEGG pathway enrichment analysis

**FIGURE 8:**
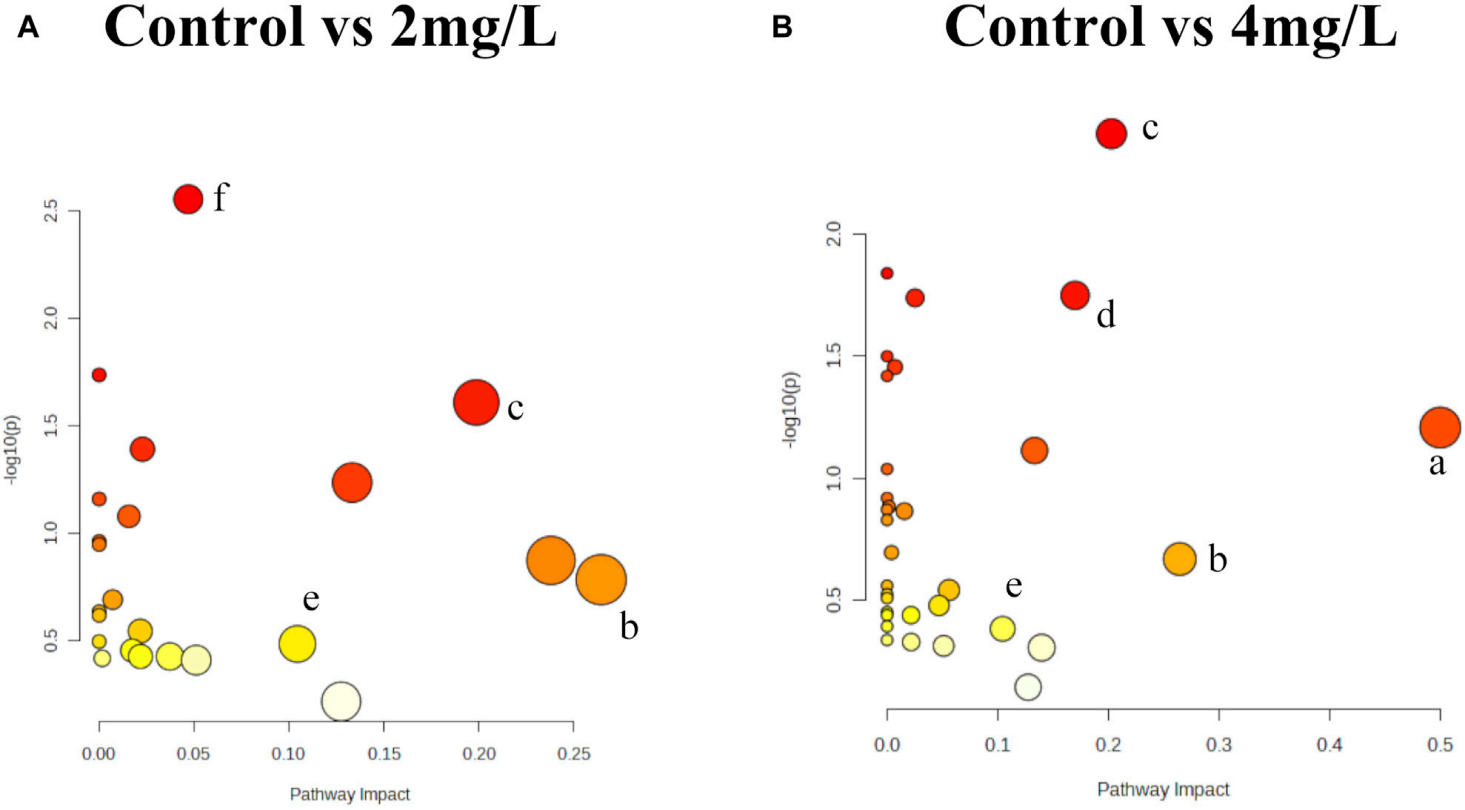
KEGG pathway analyses of differential metabolites. **(A)** Metabolic pathway based on differential metabolites in 2 mg/L (38.5 μM) Cr (VI)-exposed group. **(B)** Metabolic pathway based on differential metabolites in 4 mg/L (76.92 μM) Cr (VI)-exposed group. The X-axis illustrates the proportion of differentially expressed metabolites relative to the total number of metabolites identified within each pathway, with the size of each point indicating the abundance of differential metabolites. Notes: a: Phenylalanine, tyrosine and tryptophan biosynthesis; b: Butanoate metabolism; c: Sphingolipid metabolism; d: Glycerophospholipid metabolism; e: Cysteine and methionine metabolism; f: Lysine degradation. Bubble area is proportional to the impact of each pathway, with color denoting the significance from highest in red to lowest in white.

Moreover, we conducted KEGG pathway enrichment analysis to forecast metabolic pathways based on the metabolites exhibiting differential expression. This analysis was separately performed for the 2 mg/L (38.5 μM) group and the 4 mg/L (76.92 μM) group. Differential metabolites of the Control and 2 mg/L (38.5 μM) groups were mainly enriched in butanoate metabolism, phenylalanine metabolism, sphingolipid metabolism, synthesis and degradation of ketone bodies, steroid hormone biosynthesis, cysteine and methionine metabolism, N-Glycan biosynthesis, lysine degradation, pyrimidine metabolism, purine metabolism (Figure 8A). Differential metabolites of the Control and 4 mg/L (76.92 μM) groups were mainly enriched in Phenylalanine, tyrosine and tryptophan biosynthesis, butanoate metabolism, sphingolipid metabolism, glycerophospholipid metabolism, tyrosine metabolism, synthesis and degradation of ketone bodies, steroid hormone biosynthesis, cysteine and methionine metabolism, beta-Alanine metabolism, N-Glycan biosynthesis, lysine degradation, purine metabolism (Figure 8B). It should be noted that the differential metabolites between the 2 mg/L (38.5 μM) and 4 mg/L (76.92 μM) groups in astrocytes were mostly enriched in the sphingolipid metabolism and cysteine and methionine metabolism (Figures 8A, B).

In the examination of metabolic profiles, it was observed that the majority of altered metabolites exhibited upregulation following exposure to 2 mg/L (38.5 μM) Cr (VI) (Figure 7B), while they showed downregulation after exposure to 4 mg/L (76.92 μM) Cr (VI) (Figure 7C). These alterations were particularly enriched in pathways associated with phenylalanine, tyrosine, and tryptophan biosynthesis, butanoate metabolism, phenylalanine metabolism, sphingolipid metabolism, glycerol phospholipid metabolism, tyrosine metabolism, and cysteine and methionine metabolism. In the subsequent enrichment of metabolic pathways, it was found that the metabolic pathways of the 2 mg/L (38.5 μM) group and the 4 mg/L (76.92 μM) group are not completely consistent, with only a few parts overlapping.

Our experimental results show that under exposure to hexavalent chromium, the metabolism level of sphingosine in astrocytes significantly increased in the 2 mg/L (38.5 μM) group but decreased significantly in the 4 mg/L (76.92 μM) group. Sphingosine is a phospholipid. On the plasma membrane, sphingolipids can be converted to Sphingosine (Sph), and then Sphingosine kinase (SPHK or SK) catalyzes the production of Sphingosine 1-phosphate (S1P). The S1P signaling pathway is indispensable for orchestrating a wide array of cellular events, encompassing the survival, proliferation, differentiation, and migration of neurons, astrocytes, and microglia (Gaire and Choi, 2021). S1P plays a pivotal role in numerous biological mechanisms associated with neurodegeneration, encompassing neurotoxicity, autophagy, and neuroinflammation (Mitroi et al., 2016; Zeng et al., 2022; van Echten-Deckert, 2023). Research has found that S1P enhances cell viability by reducing mitochondrial dysfunction and cell death in neuroblastoma cells under ischemic stress. The results indicate that S1P induces mitochondrial PKC-ε translocation, alleviating mitochondrial calcium overload, mitochondrial membrane potential depolarization, and swelling, exerting neuroprotective effects during brain ischemia (Agudo-Lopez et al., 2010). Therefore, we infer that the initial increase in sphingosine content in Cr (VI)-exposed astrocytes serves a protective role for the cells, while the subsequent decrease significantly affects mitochondrial function.

Butanoate can be systemically disseminated and detected in the rat brain (Tan et al., 2014). Butanoate in the rat brain can exert neuro-protective effects against neurodegenerative disorders and improve behavioral deficits via anti-inflammatory responses (Kim et al., 2007), suggesting that it plays an important intermediary role in the alteration of neurobiological functions. Our research findings indicate that butanoate metabolism is significantly upregulated in the 2 mg/L (38.5 μM) group, but decreases in the 4 mg/L (76.92 μM) group, adding further evidence to our previous assertion: there are protective changes in cells before significant alterations in cell viability occur.

In the 4 mg/L group, there was a significant downregulation trend observed in the biosynthesis of phenylalanine, tyrosine, and tryptophan, which was the most notable change. L-phenylalanine is an essential amino acid that can be converted to tyrosine, associated with energy metabolism and immunity (Zheng et al., 2018). L-tryptophan serves as a key biomarker in inflammation and immune responses (Ma et al., 2019). In a study on the neurotoxicity induced by the co-administration of isoniazid and rifampicin, metabolomics results from mouse brain tissue revealed significant alterations in the biosynthesis of phenylalanine, tyrosine, and tryptophan (Qu et al., 2023). Additionally, the pathways of phenylalanine metabolism and tryptophan metabolism in rat brain tissue were also significantly affected under the combined influence of heavy metals (Xie et al., 2023). These pieces of evidence, combined with our data, can serve as relevant clues for further research in the future.

In addition, our experimental results also show that the content of methionine has significantly changed, and cysteine and methionine metabolism is affected. Methionine is one of the essential amino acid for human body, which plays three main roles in the body: firstly, providing the sulfur needed by the body, the second is to provide the necessary methyl groups for the body, and the third is as the precursor of sulfur-containing amino acids, cysteine, glutathione and taurine. Methionine is essential for brain function and acts as a potent antioxidant due to the sulfhydryl groups it contains, which scavenge free radicals and possess antioxidant properties. Consequently, methionine plays a pivotal role in controlling the levels of reactive oxygen radicals, both directly and indirectly (Flores et al., 2013). Methionine metabolism generates cysteine, which in turn synthesizes glutathione, a crucial antioxidant molecule that participates in protein translation (Elango, 2020). Studies have shown that the prevention of risk factors involved in the metabolism of cysteine and methionine may have important preventive significance for degenerative disease, dementia and stroke (Rita et al., 2021). Additionally, in the Methionine-Homocysteine cycle, an imbalance in methionine metabolism can result in elevated levels of homocysteine (Hcy) in circulating plasma. Hcy is a sulfur-containing amino acid formed through the metabolism of methionine into cysteine. Increased plasma levels of Hcy, known as Hyperhomocysteinemia (hHcy), represent a recognized risk factor for cardiovascular and cerebrovascular diseases (Kovalska et al., 2020).

**FIGURE 9:**
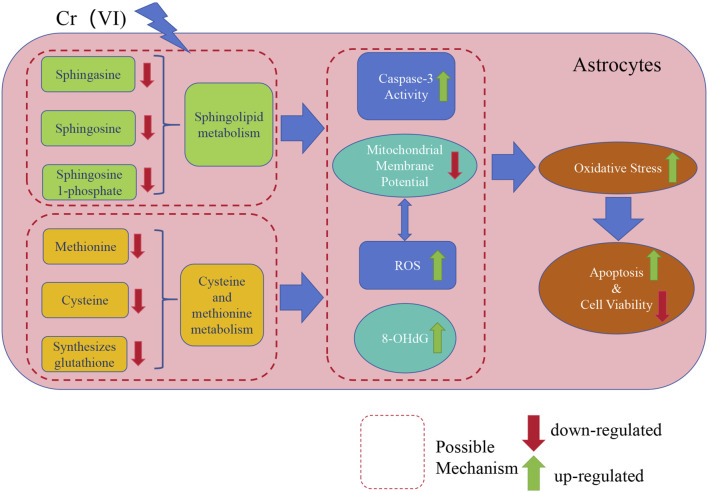
A concise mechanistic illustration delineating the study’s findings. Cr (VI) downregulates sphingolipid metabolism and the methionine-cysteine cycle, further influencing Caspase3 activity and ROS generation, leading to MMP collapse and upregulation of 8-OHdG. Simultaneously, the decrease in MMP also results in an excessive generation of ROS. This results in oxidative damage and exacerbates cell apoptosis, ultimately leading to decreased cell viability.

Finally, our study elucidated the toxic effects of Cr (VI) on astrocytes and its potential neurotoxic mechanisms, which are of significant importance in the field of neurotoxicology. Firstly, we found that oxidative stress and apoptosis induced by Cr (VI) may be meaningful pathways of neurotoxicity, providing crucial insights into the toxic mechanisms of Cr (VI) in the nervous system. Subsequently, we identified the critical roles of sphingolipid metabolism and the methionine-cysteine cycle in neurotoxicity, with sphingolipid metabolism being associated with apoptosis and the methionine-cysteine cycle playing a significant role in oxidative damage. This offers new perspectives for further research on the functions and regulation of these metabolic pathways in the nervous system. Additionally, we employed untargeted metabolomics techniques for the first time to reveal the impact of Cr (VI) exposure on the metabolic profiles of astrocytes, providing new methods and insights for the identification and assessment of neurotoxic substances. The relevant mechanism is illustrated in Figure 9.

Therefore, our study not only deepens the understanding of Cr (VI) neurotoxicity but also provides important insights for the development of neuro-protective strategies against Cr (VI) exposure in the future. Our findings underscore the potential neuro-health risks associated with environmental and occupational exposure to Cr (VI) and offer new perspectives and directions for the study of neurotoxic mechanisms.

However, it must be acknowledged that our study has certain limitations, such as a relatively small sample size and a lack of *in vivo* experiments. In future research, these experiments will be designed comprehensively to better investigate the toxic mechanisms of Cr (VI) and to discover potential biomarkers.

**TABLE 1:** The model parameters of PLS-DA in different comparative groups.

	R2X	R2Y	Q2
1 mg/L VS Control	0.607	0.647	0.47
2 mg/L VS Control	0.716	0.978	0.887
4 mg/L VS Control	0.747	0.995	0.462

## Data Availability

The original contributions presented in the study are included in the article/Supplementary Material, further inquiries can be directed to the corresponding author.
