# Supplementary material for: Study on the metabolic effects of hexavalent chromium [Cr (VI)] on rat astrocytes using un-targeted metabolomics
Source: Front Mol Biosci. 2024 Jul 5;11:1372783. doi: 10.3389/fmolb.2024.1372783 (PMC11257857; doi:10.3389/fmolb.2024.1372783)
Supplement: Supplementary file 1 [file DataSheet1.pdf]

Table S1. Cellular metabolites of astrocytes treated with 1mg/L Cr(VI).

	logFC	P.Value
(10S)-Juvenile hormone III diol	-12.541247	2.99E-18
2-Undecyl-4(1H)-quinolinone N-oxide	-12.674148	1.03E-17
Lansioside A	13.205055	4.75E-16
Gitogenin	-12.667658	1.22E-12
O-Arachidonoyl Ethanolamine	4.6395836	8.16E-09
gamma-tocopherol	15.856806	1.97E-05
Cyclandelate	1.1671019	2.60E-05
2-methoxy-5Z-hexadecenoic acid	10.951837	4.73E-04
Schottenol3-glucoside	10.569567	4.82E-04
Stearamide	-10.574749	5.30E-04
N-Hydroxyl-tryptamine	-12.631063	6.30E-04
Penicillin G	-11.183838	6.73E-04

Table S2. Cellular metabolites of astrocytes treated with 2mg/L Cr(VI)

Symbol	logFC	P.Value
O-Succinyl-L-homoserine	16.951344	8.87E-21
3-Guanidinopropanoate	23.019062	2.44E-20
(±)-2-Methylthiazolidine	-16.049786	4.06E-20
2-Succinyl-5-enolpyruvyl-6-hydroxy-3-cyclohexene-1-carboxylate	15.640608	1.77E-19
2-Hexaprenyl-6-methoxyphenol	12.76672	4.41E-19

m-Coumaric acid	15.209518	6.14E-19
Tranexamic acid	14.961575	1.21E-18
Ciclesonide	13.203079	2.78E-17
MG(0:0/18:0/0:0)	13.607804	3.42E-17
Stearamide	13.663863	3.96E-17
Momordol	14.389568	8.16E-17
Adenylosuccinic acid	12.914804	2.29E-16
farnesyl triphosphate	12.69128	8.59E-16
Sphinganine	16.427504	1.13E-15
8-HpODE	12.805538	3.72E-15
Acetyl-DL-Leucine	14.583363	3.72E-15
Chrycorin	14.181469	4.39E-15
Triprolidine	15.020573	1.08E-14
23-Acetoxysoladulcidine	13.730757	1.79E-14
N-Succinyl-L-glutamate	14.05089	3.19E-12
Coutaric acid	15.1712055	3.23E-12
all-trans-pentaprenyl diphosphate	13.75252	6.04E-12
Cilazapril	13.081579	2.72E-11
N-Caffeoyltryptophan	13.886387	5.13E-11
Thapsigargin	15.210481	5.77E-11
Merodesmosine	15.589229	8.49E-11
2,5-Dichloro-4-oxohex-2-enedioate	-1.0047842	1.31E-08
O-Arachidonoyl Ethanolamine	-3.7316341	3.50E-08

1,2,4-Nonadecanetriol	1.4517198	7.61E-08
L-Lysine	1.1616927	1.96E-07
DL-pipecolic acid	1.123415	2.61E-07
Cyclandelate	-1.5778883	3.25E-07
D-Pantothenic acid	-1.4914167	4.57E-07
trans-Hexadec-2-enoyl carnitine	-1.0673592	5.49E-07
Cyclocalamin	-1.1703697	1.07E-06
(R)-(+)-2-Pyrrolidone-5-carboxylic acid	1.3932627	1.21E-06
Calystegine C1	1.2257891	1.72E-06
Methyl N-methylanthranilate	1.0880723	1.89E-06
Silver sulfadiazine	-1.215691	5.52E-06
Methacholine	1.0016066	5.61E-06
Elaidic carnitine	1.5316908	6.97E-06
1-Fluorocyclohexadiene-cis,cis-1,2-diol	1.0416417	7.29E-06
NADH	2.475616	1.33E-05
Androstenedione	-1.6204784	1.82E-05
LysoPC(0:0/18:0)	1.10703	2.67E-05
gamma-tocopherol	-14.742898	3.96E-05
Methenamine	-2.615919	3.98E-05
(22E, 24x)-Ergosta-4,6,8,22-tetraen-3-one	-2.2239804	4.69E-05
Methionine	-1.2842919	5.12E-05
Farnesylcysteine	-1.8227357	5.17E-05

N-gamma-L-Glutamyl-L-methionine	-1.6109382	5.66E-05
L-Carnitine	-1.4182196	7.01E-05
Propylene glycol stearate	1.5399764	1.23E-04
Tridihexethyl	1.0316108	1.52E-04
Pentachlorophenol	12.332824	1.68E-04
27-Nor-5b-cholestane-3a,7a,12a,24,25-pentol	1.5547534	2.57E-04
Bacteriochlorophyllide a	11.579734	3.36E-04
Oleamide	1.2459369	3.58E-04
Sanguisorbic acid dilactone	-11.825819	3.65E-04
Sphingosine	1.2927346	3.68E-04
Valyl-Proline	-12.332281	3.76E-04
Isopentyl beta-D-glucoside	-11.608271	3.82E-04
L-Cyclo(alanylglycyl)	13.524846	3.83E-04
Phenylethylamine	-13.62837	3.84E-04
Phenylalanyl-Isoleucine	11.845933	3.91E-04
3-Hydroxymethylantipyrine	-16.765814	4.32E-04
4-Guanidinobutanamide	-15.574448	4.34E-04
MG(18:0e/0:0/0:0)	1.1615827	4.38E-04
LysoPC(20:3(8Z,11Z,14Z))	10.616578	5.20E-04
alpha,alpha'-diethyl-4,4'-bis(2-propynyloxy)stilbene	10.3939705	5.21E-04
Hypoxanthine	-14.559782	5.26E-04
Valyl-Alanine	-12.165362	6.33E-04
Indolelactic acid	11.737035	6.67E-04

(+/-)-3-(Methylthio)heptanal	11.237912	6.74E-04
Succinoadenosine	11.347783	6.75E-04
Gentamicin	10.999651	6.76E-04
Sulfasalazine	10.766298	6.83E-04
Lithocholic acid taurine conjugate	13.424458	6.85E-04
Hordatine A	11.377881	6.86E-04
Avermectin A2a monosaccharide	11.757973	6.88E-04
Ursodeoxycholic acid 3-sulfate	10.938756	6.92E-04
Malonyl-CoA semialdehyde	10.827146	6.99E-04
dolichyl diphosphate	11.125887	7.01E-04
Trimeprazine	10.92959	7.03E-04
Putranjivain A	10.330282	7.05E-04
Marimastat	11.49641	7.05E-04
Acetoacetyl-CoA	10.210889	7.11E-04
Pithecelloside	10.732634	7.12E-04
Furfuryl thioacetate	12.718584	7.19E-04
Medicarpin 3-O-glucoside-6'-malonate	10.226868	7.28E-04
Penciclovir	12.886234	7.31E-04
Platydesmine	12.006893	7.51E-04
Modafinil	12.614625	7.61E-04
(3R)-3,4-Dihydroxy-3-(hydroxymethyl)butanenitrile 4-glucoside	11.338411	7.63E-04
Alloxan	12.097462	7.63E-04

Diphenoxylate	10.645226	7.83E-04
Lonchocarpenin	10.679049	7.92E-04
Harmanine	10.198395	7.98E-04
Pubescenol	10.763975	8.02E-04
S-Propyl-L-cysteine	12.350304	8.06E-04
Chromanol 293B	12.660721	8.24E-04
4-Amino-2-hydroxylamino-6-nitrotoluene	12.1874895	8.27E-04
Oxyphencyclimine	10.992294	8.29E-04
Iprobenfos	11.58532	8.35E-04
1D-1-Guanidino-3-amino-1,3-dideoxy-scyllo-inositol	10.106315	8.61E-04
Clodronate	-1.0149615	0.001186306
4-Aminobenzenesulfonate	12.059742	0.001255762
Guanethidine	-1.2687768	0.001322463
Penicillin G	2.960616	0.00146795
Propofol	-1.021692	0.00302292
6-dehydrotestosterone 17-glucosiduronic acid	-10.6404705	0.003255254
Palmitoyl-EA	12.489076	0.003831247
(3R,7R)-1,3,7-Octanetriol	-1.4486642	0.003950073
Armillane	-10.966543	0.004409185
3-(2-Furanyl)-2-propenal	-12.654158	0.004419042
(R)-2-Hydroxysterculic acid	9.757255	0.006047083
S-Glutathionyl-L-cysteine	-9.878135	0.006147578
Cer(d18:0/23:0)	-8.986153	0.007161863

Dehydrogriseofulvin	9.134225	0.007217885
Momordicoside I	-1.0721577	0.007225396
Adenosine	-2.624575	0.007847484
MG(24:1(15Z)/0:0/0:0)	1.1102608	0.009164161
Methyl 3-(2,3-dihydroxy-3-methyl butyl)-4-hydroxybenzoate	-10.296055	0.009653885
Indoleacetaldehyde	9.404554	0.010337471
Tenuazonic acid	-9.472196	0.011707553
Leucyl-Arginine	9.426252	0.012508351
beta-Solanine	-1.2260324	0.01271037

Table S3. Cellular metabolites of astrocytes treated with 4mg/L Cr(VI).

Symbol	logFC	P.Value
Sanguisorbic acid dilactone	14.10081	4.66E-20
(±)-2-Methylthiazolidine	16.20675	5.17E-20
Tetrahydropentoxyline	-14.8684	3.46E-19
Riboflavin cyclic-4',5'-phosphate	-14.507	3.81E-19
2-Hexaprenyl-6-methoxyphenol	-12.6205	1.14E-18
Tranexamic acid	-16.0164	1.46E-18
Phenylalanyl-Isoleucine	-13.9621	1.59E-18
L-Cyclo(alanylglycyl)	-15.9551	2.75E-18
Oxypinnatanine	-16.442	5.57E-18
Avermectin B1a	-12.6342	9.51E-18

Adlupone	-12.7376	1.73E-17
(R)-2-Hydroxysterculic acid	-14.0632	1.90E-17
Silver sulfadiazine	-14.0908	4.89E-17
Stearamide	-13.5177	5.72E-17
MG(0:0/18:0/0:0)	-13.4616	5.85E-17
Triamcinolone hexacetonide	-12.0664	6.97E-17
2-Undecyl-4(1H)-quinolinone N-oxide	-15.8583	8.20E-17
Bacteriochlorophyllide a	-13.6229	8.70E-17
8-HpODE	-12.8866	9.37E-17
Momordol	-14.2434	1.12E-16
Withaperuvin H	-13.6931	1.19E-16
Palmitoyl-EA	-17.6263	1.34E-16
Adenylosuccinic acid	-12.7686	1.96E-16
Cephapirin	13.76788	3.00E-16
Oxyphencyclimine	-13.2347	3.19E-16
(E)-5-(3,4,5,6-Tetrahydro-3-pyridylidenemethyl)-2-furanmethanol	-15.3796	3.71E-16
farnesyl triphosphate	-12.5451	1.03E-15
Tetracosahexaenoic acid	-13.9515	1.20E-15
Sphinganine	-16.2813	1.42E-15
Monoolein	-13.0436	1.59E-15
Chrycorin	-14.0353	4.90E-15
Smilagenin	-14.0322	8.49E-15
alpha,alpha'-diethyl-4,4'-bis(2-propynyloxy)stilbene	-12.3142	9.05E-15

23-Acetoxysoladulcidine	-13.5846	2.13E-14
Inosine 2'-phosphate	15.41438	5.35E-14
Demecarium	-14.1119	8.68E-14
Pyrimidodiazepine	-14.4606	9.67E-14
3-Hydroxymugineic acid	-16.0529	1.34E-13
Atracurium	-16.9079	1.57E-13
LysoPC(20:3(8Z,11Z,14Z))	-12.5876	1.89E-13
Ursodeoxycholic acid 3-sulfate	-16.1111	2.36E-13
Phenylacetylglycine	-15.2797	2.76E-13
1-Tridecene-3,5,7,9,11-pentayne	-14.949	1.10E-12
N-Succinyl-L-glutamate	-13.9047	3.46E-12
Coutaric acid	-15.025	3.71E-12
all-trans-pentaprenyl diphosphate	-13.6063	7.24E-12
N-Caffeoyltryptophan	-13.7402	6.02E-11
Thapsigargin	-15.0643	6.64E-11
Thioridazine	-14.043	8.76E-11
Merodesmosine	-15.443	9.72E-11
1,2,4-Nonadecanetriol	-1.97081	1.18E-09
Diisobutyl phthalate	1.225258	1.91E-09
Propylene glycol stearate	-2.16498	2.13E-09
Methacholine	-1.8968	2.71E-09
L-Tyrosine	-1.60056	3.68E-09
Chondroitin	-1.93258	7.68E-09

m-Coumaric acid	-1.18019	8.07E-09
2,5-Dichloro-4-oxohex-2-enedioate	1.400553	9.32E-09
Elaidic carnitine	-2.72721	1.20E-08
Methyl N-methylanthranilate	-1.51414	1.26E-08
Abietic acid	1.08467	1.39E-08
1-Fluorocyclohexadiene-cis,cis-1,2-diol	-1.41364	1.56E-08
3-Hydroxymethylantipyrine	-1.27815	2.03E-08
Cyclandelate	1.795583	4.46E-08
sn-glycero-3-Phosphocholine	2.282206	5.35E-08
D-Pantothenic acid	-1.66595	6.59E-08
4-Hydroxycoumarin	1.161652	7.43E-08
LysoPE(20:4(8Z,11Z,14Z,17Z)/0:0)	1.179621	1.04E-07
Cyclocalamin	1.569874	1.10E-07
dodecanamide	1.11258	1.13E-07
Indoleacrylic acid	-1.23403	1.54E-07
trans-Hexadec-2-enoyl carnitine	1.366396	2.53E-07
LysoPC(0:0/18:0)	-1.46517	4.70E-07
O-Arachidonoyl Ethanolamine	2.575292	5.05E-07
1-Octadecanamine	1.10986	6.37E-07
LysoPE(22:5(7Z,10Z,13Z,16Z,19Z)/0:0)	1.415526	7.36E-07
(2R,6x)-7-Methyl-3-methylene-1,2,6,7-octanetetrol 2-glucoside	1.484932	1.62E-06
LysoPC(16:0)	-1.70524	1.79E-06
PE(22:6(4Z,7Z,10Z,13Z,16Z,19Z)/0:0)	1.188037	2.09E-06

Androstenedione	2.066077	2.49E-06
Anigorufone	-1.75956	2.66E-06
LysoPE(24:6(6Z,9Z,12Z,15Z,18Z,21Z)/0:0)	1.197655	2.78E-06
Oleamide	-2.21537	3.25E-06
Sphingosine	-2.34284	3.82E-06
Threoninyl-glutamate	-1.03771	4.17E-06
Adenosine	3.121413	4.86E-06
LysoPC(14:0)	-1.05515	5.63E-06
(R)-(+)-2-Pyrrolidone-5-carboxylic acid	-1.95234	7.40E-06
Leucyl-glutamate	-1.34205	9.23E-06
Cer(d18:0/16:0)	1.36339	1.01E-05
LysoPC(16:1(9Z))	-1.30288	1.20E-05
Threoninyl-Glycine	1.325839	1.41E-05
Methenamine	3.147302	1.44E-05
Dihydrozeatin	1.053969	2.00E-05
Guanethidine	1.607337	2.06E-05
Methionine	1.424394	2.14E-05
N-Jasmonoylisoleucine	1.013447	3.16E-05
2-Hydroxy-dAMP	-1.45998	3.89E-05
MG(18:0e/0:0/0:0)	-1.29359	4.11E-05
Dinitolmide	1.227607	4.17E-05
gamma-tocopherol	13.38731	8.78E-05
O-Succinyl-L-homoserine	-2.5179	1.27E-04

Clupanodonic acid	1.478748	1.43E-04
Sucralose	12.77781	1.43E-04
Benzaldehyde	-12.1539	1.65E-04
LysoPC(22:5(7Z,10Z,13Z,16Z,19Z))	1.006825	1.89E-04
Neoabietic acid	1.314849	1.96E-04
Allylestrenol	-11.5185	2.77E-04
Clodronate	1.362705	2.97E-04
N-Acetylmuramic acid 6-phosphate	11.41401	3.01E-04
Phenylacetonitrile	-12.7267	3.06E-04
Leucyl-Arginine	-11.9565	3.20E-04
2,4,12-Octadecatrienoic acid isobutylamide	11.34469	3.27E-04
7alpha,25-dihydroxycholesterol	-1.46463	3.42E-04
Sinapoylspermine	12.4132	3.59E-04
Cucurbitacin B	12.31065	3.60E-04
L-Histidinal	12.78916	3.64E-04
1,4-beta-D-Glucan	12.16691	3.74E-04
(22E, 24x)-Ergosta-4,6,8,22-tetraen-3-one	1.904116	4.19E-04
beta-Solanine	1.075133	4.59E-04
(±)-4-Methylene-2-pyrrolidinecarboxylic acid	11.68789	5.91E-04
Penicillin G	-3.26585	7.00E-04
trans-S-(1-Propenyl)-L-cysteine	-12.8367	7.18E-04
Indolelactic acid	-11.5908	7.32E-04
Prednicarbate	1.043818	7.32E-04

Succinoadenosine	-11.2016	7.43E-04
Lithocholic acid taurine conjugate	-13.2783	7.44E-04
Avermectin A2a monosaccharide	-11.6118	7.55E-04
Sulfasalazine	-10.6201	7.55E-04
Hordatine A	-11.2317	7.56E-04
Tomatoside A	-10.6608	7.67E-04
LysoPE(0:0/18:0)	-10.1951	7.68E-04
Malonyl-CoA semialdehyde	-10.6809	7.73E-04
dolichyl diphosphate	-10.9797	7.73E-04
Marimastat	-11.3502	7.76E-04
Trimeprazine	-10.7834	7.76E-04
Putranjivain A	-10.1841	7.84E-04
Furfuryl thioacetate	-12.5724	7.84E-04
Pithecelloside	-10.5864	7.88E-04
Malonylcarnitine	-12.8413	7.89E-04
Acetoacetyl-CoA	-10.0647	7.91E-04
Medicarpin 3-O-glucoside-6'-malonate	-10.0807	8.10E-04
Modafinil	-12.4684	8.24E-04
(3R)-3,4-Dihydroxy-3-(hydroxymethyl)butanenitrile 4-glucoside	-11.2019	8.32E-04
Alloxan	-11.9513	8.37E-04
Coroloside	-11.0896	8.48E-04
Diphenoxylate	-10.499	8.60E-04
N1,N5,N10-Triferuloyl spermidine	-10.5035	8.64E-04

L-Arginine phosphate	-10.3363	8.65E-04
S-Propyl-L-cysteine	-12.2041	8.69E-04
Schleicherastatin 3	-10.8108	8.75E-04
2-Hydroxyfelbamate	-10.7161	8.76E-04
Pentachlorophenol	-11.7767	8.84E-04
Chlorpyrifos	-10.7034	8.88E-04
Gambogic acid	-10.4031	8.90E-04
Pubescenol	-10.6178	8.91E-04
Chromanol 293B	-12.5145	8.93E-04
Naftifine	-10.0169	8.94E-04
Iprobenfos	-11.4391	9.26E-04
1D-1-Guanidino-3-amino-1,3-dideoxy-scyllo-inositol	-9.96012	9.43E-04
Tyrosyl-glutamate	1.013392	1.06E-03
LysoPC(18:1(11Z))	-1.00585	1.12E-03
Cysteinyl-Alanine	-11.5257	1.15E-03
Telaprevir	1.5143	1.61E-03
4-Hydroxy-5-methyl-3(2H)-thiophenone	-12.0107	0.001761
7-(4-Hydroxyphenyl)-1-phenyl-4-hepten-3-one	1.227029	1.84E-03
Ciclesonide	-1.86256	2.35E-03
Fulvestrant	-10.6812	2.67E-03
Istamycin FU-10	-9.78057	3.98E-03
(2S,4S)-Pinnatanine	-9.88855	4.01E-03
Inosine	12.8699	4.06E-03

4-Aminobenzenesulfonate	-11.3366	4.21E-03
Benzoquinoneacetic acid	-11.1107	0.004278
alpha-Methylstyrene	1.050176	4.34E-03
3-(2-Furanyl)-2-propenal	-10.7135	0.005182
Isomorellic acid	9.431761	5.53E-03
Sulfadiazine	-9.58672	5.86E-03
Indoleacetaldehyde	-9.54638	6.32E-03
Phytosphingosine	-9.80864	6.79E-03
Armillane	9.943463	0.007171
2-Hydroxy-dATP	1.42601	0.007338
Dihydroneopterin phosphate	10.36237	0.008121
3-(3'-Methylthio)propylmalic acid	1.001511	0.008515
Spermine	12.66986	8.78E-03
Avenestergenin A1	9.76718	9.29E-03
Abacavir	9.698072	1.01E-02
Prenyl-L-cysteine	-9.5561	1.08E-02
(R)-Dihydromaleimide	10.13052	0.010799
Saprisartan	-8.67101	1.11E-02
L-Valine	-12.5719	0.012332
Valyl-Alanine	9.89922	1.25E-02
Hypoxanthine	1.006582	1.33E-02
alpha-Butyl-omega-hydroxypoly(oxyethylene) poly(oxypropylene)	-9.174	1.50E-02
Calystegine C1	-8.327	1.51E-02

Farnesylcysteine	8.582566	1.60E-02
3-Guanidinopropanoate	-10.9239	1.69E-02
NADH	-10.2604	1.70E-02
Gentamicin	-8.77612	1.70E-02
MG(24:1(15Z)/0:0/0:0)	-7.99309	1.82E-02
Morphinone	-8.63549	1.97E-02
Norepinephrine sulfate	-7.78538	0.020294
Cilastatin	-8.27965	2.06E-02
Tridihexethyl	-7.80438	2.07E-02
PE(15:0/20:4(5Z,8Z,11Z,14Z))	-11.0705	2.08E-02
1-Methylpyrrolinium	9.763985	2.22E-02
Imidazoleacetic acid ribotide	-7.58543	2.39E-02
Harmanine	-7.86647	2.83E-02
